# Comparison of Three Methods for the Estimation of Pineal Gland Volume Using Magnetic Resonance Imaging

**DOI:** 10.1100/2012/123412

**Published:** 2012-04-19

**Authors:** Niyazi Acer, Ahmet Turan Ilıca, Ahmet Tuncay Turgut, Özlem Özçelik, Birdal Yıldırım, Mehmet Turgut

**Affiliations:** ^1^Department of Anatomy, School of Medicine, Erciyes University Kayseri, Turkey; ^2^Department of Radiology, Gülhane Military Medical School, Ankara, Turkey; ^3^Department of Radiology, Ankara Training and Research Hospital, Ankara, Turkey; ^4^Department of Emergency, Muğla State Hospital, Muğla, Turkey; ^5^Department of Neurosurgery, School of Medicine, Adnan Menderes University Aydın, Turkey

## Abstract

Pineal gland is a very important neuroendocrine organ with many physiological functions such as regulating circadian rhythm. Radiologically, the pineal gland volume is clinically important because it is usually difficult to distinguish small pineal tumors via magnetic resonance imaging (MRI). Although many studies have estimated the pineal gland volume using different techniques, to the best of our knowledge, there has so far been no stereological work done on this subject. The objective of the current paper was to determine the pineal gland volume using stereological methods and by the region of interest (ROI) on MRI. In this paper, the pineal gland volumes were calculated in a total of 62 subjects (36 females, 26 males) who were free of any pineal lesions or tumors. The mean ± SD pineal gland volumes of the point-counting, planimetry, and ROI groups were 99.55 ± 51.34, 102.69 ± 40.39, and 104.33 ± 40.45 mm^3^, respectively. No significant difference was found among the methods of calculating pineal gland volume (*P* > 0.05). From these results, it can be concluded that each technique is an unbiased, efficient, and reliable method, ideally suitable for in vivo examination of MRI data for pineal gland volume estimation.

## 1. Introduction

The human pineal gland, a part of the diencephalon, is a small neuroendocrine organ that has a function in the circadian rhythm by the secretion of melatonin neurohormone [1]. It is a circumventricular organ because of its deep location in the subarachnoid cistern surrounding the surface of the third ventricle [2, 3]. Anatomically, the pineal gland is a rounded or crescent-shaped structure like a pine cone and it is attached by the stalk to the diencephalon and the stalk lines the pineal recess whose inferior lip links the pineal gland to the posterior commissure, and superior lip to the habenular commissure [4].

Stereological methods using the Cavalieri principle have been widely applied on magnetic resonance imaging (MRI) sections to estimate volume of brain and internal brain compartments. Researchers have employed these techniques to obtain volume estimations of various brain structures, including hippocampus, temporal lobe, Broca's area, brain ventricles, cerebellum, and cerebral hemisphere [5–9].

There are several packages that have been developed for volume estimation such as Analyze and Image J. This software has ROI function based on manual techniques. Manual techniques such as planimetry or tracing methods require the investigator to delineate a brain region based on reliable anatomical landmarks, whilst the software package provides information on volume. Tracing methods require the investigator to trace the brain region of interest (ROI) using a mouse-driven cursor throughout a defined number of MRI sections [10]. The cut surface areas, determined by pixel counting within the traced region, are summed and multiplied by the distance between the consecutive sections traced to estimate the total volume.

Planimetric techniques are still time consuming and costly and are not accepted in clinical practice. Therefore, the point-counting technique used in this and previous studies makes volume estimation easier and quicker than manual techniques [6, 7, 10, 11].

Although pineal gland weight and volume vary greatly in respect of time, age, and physiological condition, the mean weight of the adult human pineal gland is generally 50 to 150 milligrams [12]. It has been stated that the pineal gland grows in size from birth until two years of age and then remains constant between 2 to 20 years of age [13]. Formerly, it was believed that the pineal gland played an important functional role in the onset of puberty [14, 15]. Some autopsy studies have reported that the average size of the pineal gland is 7.4 mm in length, 6.9 mm in width, and 2.5 mm in height [16]. Interestingly, Tapp and Huxley [17, 18] reported a gradual increase in the size of the pineal gland from puberty to old age in humans.

Recently, radiological studies of the pineal gland have been mainly conducted by computed tomography (CT) on pineal calcification over different populations of healthy subjects [19, 20]. There have been a few studies about pineal volume estimation using different methods such as elliptic approaches and ROI on MRI [21–23], but, to the best of our knowledge, there has been no study using stereological methods. Therefore, this study aimed to measure pineal gland volume based on 3.0-T MRI data using three different stereological methods.

## 2. Materials and Methods

### 2.1. Patient Population

The study group consisted of 35 women (age range: 11–75 years, average 44.05 ± 17.28 years) and 27 men (age range: 10–73 years, average 32.29 ± 18.58 years) who had undergone cranial MRI studies at Gülhane Military School of Medicine, Department of Radiology, Ankara, Turkey. The radiology reports and electronic medical records of each patient were retrospectively reviewed to determine the indication for MRI and to record any history of surgery to the epiphyseal area, or symptoms referable to the pineal gland. For the purposes of this study, patients were excluded if there was a history of pineal tumor, cyst, or dysfunction, if there was any brain abnormality adjacent to the pineal gland, or if the required images were missing or destroyed. 

We obtained informed written consent from each subject and approval from the Local Ethics Committee of Gülhane Military School of Medicine before the initiation of this retrospective study.

### 2.2. Image Acquisition

All images were obtained with a Philips Achieva (Philips Medical Systems, Best, The Netherlands) 3.0-(release 2.6.3.series) t MRI magnet. Thin section MRI data were obtained using sagittal 3D T1-weighted turbo field echo (TFE) sequence (TR/TE = 8.3 ms/3.9 ms; voxel size = 1 mm × 1 mm × 1 mm; FOV = 240 mm; matrix size = 224 × 256; flip angle (FA) = 8°; slice thickness = 1 mm without gap; NSA = 2).

In this study, the pineal boundary was exactly identified on the sagittal sections taken in addition to coronal and axial views. We used T1-weighted images because there was a better contrast resolution for the gland on T1-weighted sequences than on T2-weighted sequences.

### 2.3. Volume Estimation Using Different Methods

Planimetry and point counting are two different methods for volume estimation based on the Cavalieri principle. From these, planimetry which involves manually tracing the boundaries of objects of interest on images of sections is the most commonly used technique for estimation of volume, while the point-counting method use a regular grid of test points [8, 24]. Some software about volumetric measurements has an ROI function such as DICOM viewer. Using planimetry or point-counting technique, we can also estimate the volume of any organ using these methods.

In the current study, we used 1 mm slice thickness for three stereological methods for pineal gland volume estimation: planimetry, point counting, and ROI.

#### 2.3.1. Image Analysis for ROI

**Figure 1:**
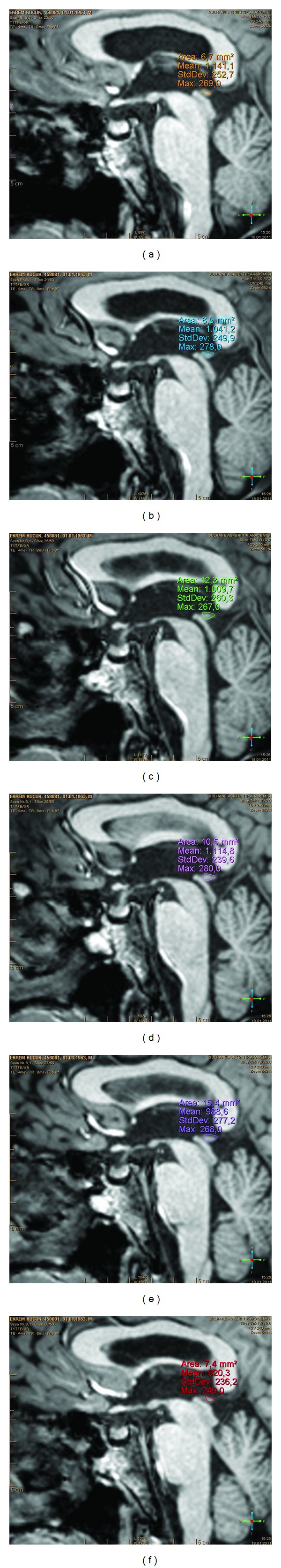
A sagittal MRI with an ROI contour on it for the estimation of pineal gland volume from first to last section.

The ROI of each pineal gland was measured by Mediplus DICOM viewer (TURMAP, 2006, v.2.6.0). All glands were traced blind by two expert radiologists independently (ATI, ATT). They identified slices displaying pineal tissue and manually defined ROIs, including all the gland tissue (Figure 1). The volume of the pineal gland was calculated by multiplying the summed pixel cross-sectional areas by slice thickness.

 Firstly, the saved T1-weighted images were opened into 1 mm sagittal sections. The ROI areas were measured with Mediplus DICOM viewer. Then, the images were displayed on a 21-inch monitor and each ROI was traced manually using a digitizing tablet. Lastly, pineal gland volume was obtained by summing the cut surface area from all of the ROIs and multiplying by the sum of the slice thickness.

Using this technique, there are some differences between observers. So we calculated both interrater and intrarater reliability coefficients. Interrater reliability was computed from measures by two radiologists who traced all pineal glands.

#### 2.3.2. Stereological Approaches

**(a) Point-Counting Method**

The Cavalieri method in combination with point counting requires beginning from a uniform random starting within the sectioning interval, a structure of interest is exhaustively sectioned with a series of parallel plane probes a constant distant apart. An unbiased estimate of volume is obtained by multiplying the total area of all sections through the structure by sectioning interval *t* as follows:

(1) $$\text{est}V=t\times \left(a_{1}+a_{2}+\cdots +a_{n}\right),$$

where *a*_1_, *a*_2_,…, *a*_*n*_ show the section areas and *t* is the sectioning interval [25, 26].

The point-counting method uses a regular grid of test points to superimpose each MRI. After each superimposition, the number of test points hitting the structure of interest is counted on each section. If we use regular grid of test points we can estimate volume following the formula;

(2) $$\text{est}V=t\times \left(\frac{a}{p}\right)\times \left(p_{1}+p_{2}+\cdots +p_{n}\right),$$

where *p*_1_, *p*_2_,…, *p*_*n*_ show point counts and *a*/*p* represent the area associated with each test point. To avoid bias the position of the test system should be uniform randomly [25, 26].

For estimating pineal gland volume we used the MRIs of a section series for each pineal gland with slice thickness in 1 mm interval. The images were opened on computer and the transparent square grid test system with *d* = 15 mm between test points was superimposed, randomly covering the entire image frame. The modified formula used firstly by Sahin and Ergur [24] for volume estimations of radiological images using the MRI scale. The points hitting the pineal gland sectioned surface area were counted for each section and the volume of the pineal gland was estimated using the modified formula:

(3) $$V\left(pc\right)=t\times {\left[\frac{\text{su}\times d}{\text{sl}}\right]}^{2}\times \overset{}{\underset{}{\sum }}p,$$

where “*t*” is the section thickness, “su” the scale unit, “*d*” the distance between the test points of the grid, “sl” the measured length of the scale, and “$\sum_{}^{}p$” is the total number of points hitting the sectioned cut surface areas of the pineal gland.

**Error Prediction**

The coefficient of error (CE) is given below and comes from recent papers [26, 27]. The CE is computed as follows:

(4) $$\text{c}{\text{e}}^{2}\left(\tilde{V}\right)={\text{c}{\text{e}}^{2}}_{\text{CAV}}\left(\hat{V}\right)+\text{c}{{\text{e}}^{2}}_{\text{PC}}\left(\tilde{V}\right).$$

It can be shown [26] that $\text{c}{\text{e}}^{2}\left(\tilde{V}\right):$ CE of the volume estimate, $\text{c}{{\text{e}}^{2}}_{\text{PC}}\left(\tilde{V}\right):$ true mean variability due to point counting within sections, $\text{c}{{\text{e}}^{2}}_{\text{CAV}}\left(\hat{V}\right):$ true contribution of the variability among sections.

First, we need to compute the quantities *C*_0_, *C*_1_, *C*_2_, *C*_4_. Therefore, we used the following equation:

(5) $$C_{k}=\overset{n-k}{\underset{i=1}{\sum }}P_{i}P_{i+k},\;k=0,1,2,\ldots ,n-1.$$

*P*_*i*_ show the number of test points hitting a section of area. We need to compute the quantities *C*_0_, *C*_1_, *C*_2_, *C*_4_ by means of (7).

The dimensionless shape is calculated from the mean boundary length and the mean area of the sections, respectively [28]. Thus, $B\sqrt{A}$ is a dimensionless shape coefficient of the sections.

For the pineal gland, we estimated $B\sqrt{A}=5.5$ [26, 28] and the nugget variance ($\hat{\nu }$) is therefore

(6) $$\hat{\nu }=0.0724\cdot \left(\frac{B}{\sqrt{A}}\right)\times \sqrt{n}\sum Pi.$$

The variation of sections is predicted as [26],

(7) $$\text{c}{\text{e}}^{\text{2}}\left(\hat{V}\right)=a\left(q\right)\times \left(3\left(C_{0}-\hat{v}\right)-4C_{1}+C_{2}\right)\times {\left(\overset{}{\underset{}{\sum }}Pi\right)}^{-2}.$$

The accuracy of the predictor $\text{c}{\text{e}}^{\text{2}}\left(\tilde{V}\right)$ depends on the value of *q* used [29]. By (8) the estimate of the smoothness constant (*q*) becomes as following:

(8) $$q=\max\left\{0,\frac{1}{2\log\left(2\right)}\times \log\left(\frac{3\left(C_{0}-\hat{\nu }\right)-4C_{2}+C_{4}}{3\left(C_{0}-\hat{\nu }\right)-4C_{1}+C_{2}}\right)-\frac{1}{2}\right\}.$$

The coefficient depends on the fractional smoothness constant *q* of the area function, and *α*(*q*) has the following expression:

(9) $$a\left(q\right)=\frac{\Gamma \left(2q_{i}+2\right)\times \zeta \left(2q_{i}+2\right)\times \cos\left(\tau q_{i}\right)}{{\left(2\tau \right)}^{2qi+2}\times \left(1-2^{2qi-1}\right)}.$$

Γ() and *ζ*() denote the gamma function and the Riemann function, respectively [26, 29].

Having found the quantities for the variables in (5), we can determine the estimated value of the variation between sections.

From (6) the mean variability in the estimate due to point counting can be calculated:

(10) $${\text{c}{\text{e}}^{\text{2}}}_{\text{PC}}\left(\hat{V}\right)=\hat{V}\times {\left(\overset{}{\underset{}{\sum }}Pi\right)}^{-2}.$$

We used (7) for calculation CE_CAV_ value.

(11) $$\text{c}{{\text{e}}_{\text{CAV}}}^{2}\left(\hat{V}\right)=a\left(q\right)\times \left(3\left(C_{0}-\hat{v}\right)-4C_{1}+C_{2}\right)\times {\left(\sum Pi\right)}^{-2}.$$

We added CE_CAV_ and CE_PC_ for total CE.

Finally, we can now calculate the total CE of the volume estimate in (4);

(12) $$\text{c}{{\text{e}}^{2}}_{\text{TOTAL}}\left(\tilde{V}\right)=\text{c}{{\text{e}}^{2}}_{\text{CAV}}\left(\hat{V}\right)+\text{c}{{\text{e}}^{2}}_{\text{PC}}\left(\tilde{V}\right).$$

In this study, we calculated the CE values as predictive using the *R* program. Firstly, using the statistical package *R* (http://www.r-project.org/) codes were developed to calculate the contribution to the predictive CE [26, 29]. The stereological approach gives an opportunity to the researcher making appropriate changes on their sampling or estimating procedures. Therefore, the current study provides a CE of estimation for volume assessment. A CE value lower than 10% is in acceptable range [30].

**(b) Planimetry Method**

The T1 sequence was transferred to a PC and further image processing was performed using image analysis software as ImageJ. The images were displayed on a monitor with fixed contrast settings using consistent image and display levels [8, 9].

The observer who carried out the stereological volume estimates also performed the pineal gland volume estimates using planimetry. The pineal gland boundaries were manually traced on each MRI section using the computer mouse. The cross-sectional surface area was measured by means of the planimetry method using ImageJ software (http://rsbweb.nih.gov/ij/download.html).

In a sample of the data (*N* = 62), the number of sagittal slices traced per case varied from 5 to 7 (mean ± S.D. = 6.4 ± 0.2 slices).

As previously described [8, 31], the rater traced around the area of interest within each slice. The software calculated the number of pixels enclosed within the traced area and the process was repeated for each slice. Since the pixel dimension and slice thickness were known, pineal gland volume could be estimated (13):

(13) $$V\left(PL\right)=t\times \overset{}{\underset{}{\sum }}a,$$

where “*t*” is the section thickness, and $\sum_{}^{}a$ is the total sectional area of the consecutive sections millimeter square of the pineal gland.

### 2.4. Statistical Analysis

Statistical analysis was performed using SPSS 17.0 (SPSS Inc., Chicago, IL). The values of three methods are presented as mean and standard deviations (mean ± S.D.). The differences between the estimated volumes obtained by three different approaches, namely, ROI, point counting, and planimetry, were compared using Tukey's test and Bland-Altman analysis to check the methodological differences. The effect of sex on pineal gland volume was tested for using independent *t*-tests.

The correlation between age and pineal gland volume was tested for using Pearson's product correlation coefficient. The intraclass correlation coefficient (ICC), using one-way random effects analysis of variance test, was determined for the ROI volumetric assessments in order to show the inter- and intraobserver agreement. We also tested effects of age, and sex on volume measures determined by each method, and then involved age and sex as covariates in linear model.

A *P* value of <0.05 was considered as statistically significant.

## 3. Results

**Figure 2:**
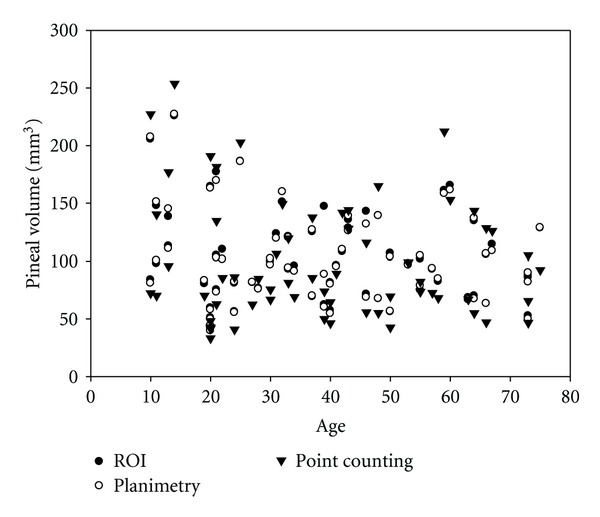
The relationship between age and average of the pineal gland volume using three methods.

No significant gender difference of pineal volume was found by Student's *t*-test for the three methods in the whole sample (*P* > 0.05; Table 1). In the whole sample, there were no significant correlations between pineal volume and age for the three methods (*r* = −0.174, −0.179, −0.163, *P* > 0.05) using Pearson's correlation test (Figure 2). The mean ± S.D. pineal gland volumes for the point-counting, planimetry, and ROI groups were 99.55 ± 51.34,102.69 ± 40.39, and 104.33 ± 40.45 mm^3^, respectively (Table 1).

 The results of pineal volume values obtained using these three methods were compared statistically. No significant difference was found for the three methods using one-way ANOVA analysis (*P* = 0.830, *F* = 0.186, *P* > 0.05; Table 2).

The mean CE for pineal gland volume estimates derived from the point-counting technique was 5.88% (Table 3).

**Figure 3:**
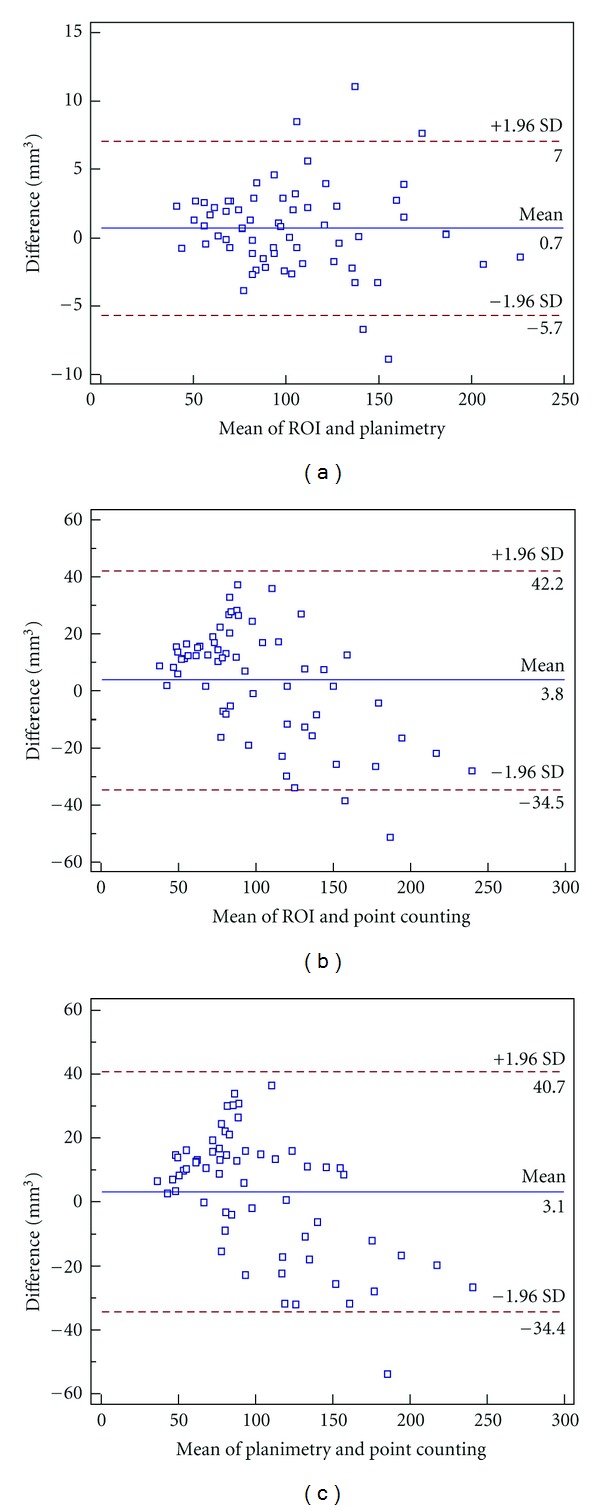
A Bland-Altman plot analysis of the pineal volume as measured by ROI, point-counting (PC) technique, and planimetric method (PL). (a) ROI versus planimetry, (b) ROI versus point counting, and (c) point counting versus planimetry.

Bland-Altman analysis showed that the 95% of the mean of volumes estimated by ROI and planimetry, ROI and point counting, and point counting and planimetry were 0.7 cm^3^, 3.8 cm^3^, and −3.1 cm^3^, respectively (Figure 3).

The point-counting technique did, however, take less time than planimetry and ROI to calculate pineal gland volume from MRIs. The mean time (± SD) needed to estimate the pineal volume using the point-counting technique, planimetry, and ROI were 0.95 ± 0.3 minutes, with a range of 0.5–1.5 minutes; 2.0 ± 1.1 minutes, with a range of 1.4–3.8 minutes; 3.5 minutes, with range of 2.8–4.3 minutes, respectively.

We examined the effects of age and sex on the pineal gland volumes determined by ROI, planimetry, and point counting, using a linear regression model and the effects of age and sex were not significant (*P* > 0.05).

For three measurements, performed on MRIs, a set of slices containing each ROI was split, at random, into two equal groups, and each was traced by two radiologists. The ICC intra-rater agreement was 0.95 (*P* < 0.001) for radiologist 1 Ahmet Turan Ilica and 0.96 (*P* < 0.001) for radiologist 2 Ahmet Tuncay Turgut. The ICC of the interrater agreenment for all ROIs was 0.94.

## 4. Discussion

The pineal gland is a solid neuroendocrine organ, located deep in the complicated pineal region of varying morphological characteristics and shape [23, 32]. Therefore, an estimation of the true pineal volume is difficult using one- or two-dimensional parameters (e.g., pineal length). Sumida et al. [13] used the ellipsoid formula for the pineal gland, measuring maximum length (*L*), height (*H*), and width (*W*). The volume (*V*) was calculated according to the formula *V* = 1/2 × *H* × *L* × *W* [13, 33].

In previous studies the pineal volume was only calculated as an estimated value which would be too approximate to represent the features of pineal gland [13, 19, 22, 34]. Using a locally developed software (BRAINS, Brain Research: Analysis of Images, Networks, and Systems), Rajarethinam et al. [21] obtained the pineal volume by blind manual tracing of the gland on T1-weighted images. Afterwards, Sun et al. [23] used T1-weighted images obtained from 3.0-T MRI scanner to calculate the pineal volume using pineal length, width, and height for the first time. In their study, Sun et al. [23] used the ellipsoid formula for pineal gland estimation, but we used stereological methods for volume estimation in this study. Schmitz et al. [19] used a semi-quantitative CT protocol to determine uncalcified pineal tissue. In their study, a total of 22 pineal gland autopsy specimens were scanned in a skull phantom with different slice thickness, and the uncalcified tissue was visually assessed using a four-point scale and was measured, and its inverse graded on a nonlinear four-point scale. Then, the sum of both scores was multiplied by the gland volume to yield the uncalcified pineal tissue [19]. On the other hand, Golan et al. [34] compared the size, weight, volume, and density of the pineal glands of 80 humans in several groups divided by age, body weight, and height. Interestingly, they found that there were no differences between morphometric structure of the gland and body weight [35]. Also, there were no age-related changes in the morphometry of the pineal gland [19]. In a previous study, Schmitz et al. [19] used planimetry of pineal outlines in either photographs or camera lucida drawings of serial histological sections for rodents using a stereological method. In our study, however, the pineal volumes of healthy young adults were obtained based on 3.0-T MRI data. We found that the effects of age and sex on the pineal gland volumes using a linear regression model and the effects of age and sex were not significant.

From a technical point of view, Rajarethinam et al. [21] obtained the volume of the pineal by blind manual tracing of the gland on T1-weighted MRIs, using locally developed software. Nölte et al. [36] estimated the pineal gland volume on MRI using the volume analysis program. Bersani et al. [22] calculated pineal gland volume on MRI sections using elliptic formula. Rajarethinam et al. [21] and Sun et al. [23] used ROI analysis for pineal gland volume estimation on MRI sections. The difference in the pineal gland volume found may be attributable to the differences methodology used in these studies.

Unfortunately, there is no consensus regarding the volume of the pineal gland in humans; some studies found that the pineal volume was small, whereas these results were in contrast with others. In a three-dimensional volumetric study using 3.0-T MRI, Sun et al. [23] reported that pineal volume was 94.2 ± 40.65 mm^3^ in healthy young adults, while Rajarethinam et al. [21] found that pineal volume was 213 mm^3^ for control subjects. Nölte et al. [36] found that the pineal gland volume was 125 ± 54 mm^3^. In the present study, the mean ± S.D pineal gland volumes for point-counting, planimetry, and ROI groups were found to be 99.55 ± 51.34, 102.69 ± 40.39, and 104.33 ± 40.45 mm^3^, respectively. These results are lower than those of Rajarethinam et al. [21] and Nölte et al. [36], but slightly higher than the Sun et al. [23] and Bersani et al. [22] results.

In a previous study, it had been stated that the pineal gland played an important role in the onset of puberty [15]. Sumida et al. [13] reported that pineal gland size increased from birth until 2 years of age and remained constant 20 years of age. In this study, the average size of the normal pineal gland was determined in children, adolescents, and adults.

Recently, MRI has provided a marked improvement in the ability to locate and characterize tissue. For instance, small noncalcified structures that cannot be detected with CT are seen clearly on MRIs, especially in the midline on sagittal sections [37]. Thus, CT and, preferably, MRI are the imaging modalities to show normal anatomy and the majority of pathologic processes in this gland. In clinical practice, an understanding of the radiological anatomy of the pineal region and its surrounding structures is crucial for evaluating the broad spectrum of radiologic pathologies that can involve the gland. Despite being a straightforward method, the contrast resolution and multiplanar imaging capability of CT is limited. On the contrary, MRI, which does not involve the use of ionizing radiation, is preferred over CT especially in children and patients requiring multiple imaging studies. MRI can be performed in any imaging plane without moving the patient. The use of MRI, with or without contrast, enables accurate anatomic delineation of the tumoral mass and the determination of the pineal gland in relation to the surrounding structures [38].

Some studies have found no correlation between pineal volume and age [13, 19] whereas others have [23]. A possible explanation for the above-mentioned contradiction about the age-related trends of pineal volume is the different methods used. The results of this study determined no significant correlations between pineal volume and age.

A few studies have compared pineal volume and a disease such as schizophrenia. Rajarethinam et al. [21] compared the volume of the pineal gland in a sample of schizophrenic patients and in normal controls, finding no significant differences between the groups. Bersani et al. [22] stated that the mean ± SD pineal volume was 64.05 ± 20.69 mm^3^ for schizophrenics and 74.62 ± 33.53 mm^3^ for controls, with a significant difference between the groups (*P* = 0.022) shown by the 2-tailed Student's *t*-test. Although there have been many studies about pineal volume, to the best of our knowledge, there has been no study to date which calculated the CE value. In this study it was determined that the mean CE for pineal gland volume estimates derived from the point-counting technique was 5.88%. However, a limitation of the study is that only healthy subjects were studied, that is, the performance of the three methods may be different under pathological conditions. In the future, we plan to investigate the effects of pathological conditions, including schizophrenia.

## 5. Conclusion

Based on our data, a point spacing of 15 mm, and a section thickness of 1 mm were considered to be ideal for estimating pineal gland volumes using stereological approaches with point-counting technique in few minutes. Stereology combined with point-counting technique is much more time efficient than manual tracing methods such as planimetry and ROI, with respect to the time taken to estimate volume of the pineal gland on MRI. Furthermore, it can be concluded from the results of this study that each technique can be used but the point-counting method is an unbiased, efficient, and reliable method and ideally suitable for in vivo examination of MRI data for pineal gland volume estimation. 

## Figures and Tables

**Table 1:** Mean volume estimates for the pineal gland for men, women, and total.

	Men (*n* = 27)	Women (*n* = 35)	Total (*n* = 62)	*P*
	Mean ± SD	Mean ± SD	Mean ± SD	
ROI	103.54 ± 45.35	105.90 ± 37.16	104.33 ± 40.45	0.93
Planimetry	102.44 ± 45.44	102.87 ± 37.81	102.69 ± 40.39	0.86
Point counting	101.36 ± 56.75	98.24 ± 44.87	99.55 ± 51.34	0.79

**Table 2:** The result of analysis of variance along with postrhoc test (Tukey).

	Mean differences	Standard error (SE)	Confidence interval (CI)		*P*
			Lower	Upper	
ROI—Planimetry	1.64	7.96	−17.18	20.46	0.97
ROI—Point counting	4.78	7.96	−14.04	23.61	0.82
Planimetry—Point counting	−3.14	7.96	−21.96	15.68	0.91

**Table 3:** Coefficient of error values for point counting (CE_PC_), Cavalieri (CE_CAV_) and total (CE_Total_).

	Minimum	Maximum	Mean	Std. Deviation
CE_PC_	0.85	11.65	4.61	2.60
CE_CAV_	1.76	5.72	3.31	0.91
CE_Total_	2.07	12.07	5.88	2.27

## Abbreviations

ROI: Region of interest
TFE: Turbo field echo
FA: Flip angle.
